# Cardiac amyloidosis screening using a relative apical sparing pattern in patients with left ventricular hypertrophy

**DOI:** 10.1186/s12947-021-00258-x

**Published:** 2021-08-23

**Authors:** Yasuhisa Nakao, Makoto Saito, Katsuji Inoue, Rieko Higaki, Yuki Yokomoto, Akiyoshi Ogimoto, Moeko Suzuki, Hideo Kawakami, Go Hiasa, Hideki Okayama, Shuntaro Ikeda, Osamu Yamaguchi

**Affiliations:** 1 Department of Cardiology, Kitaishikai Hospital, Higashiozu 1563-1, Ozu, Ehime 795-8505 Japan; 2 Department of Cardiology, Pulmonology, Nephrology and Hypertension, Ehime University Graduate School of Medicine, Toon, Japan; 3 Department of Cardiology, Uwajima City Hospital, Uwajima, Japan; 4 Department of Cardiology, Ehime Prefectural Imabari Hospital, Imabari, Japan; 5 Department of Cardiology, Ehime Prefectural Central Hospital, Matsuyama, Japan

**Keywords:** Apical sparing, Cardiac amyloidosis, Strain, Score, Screening

## Abstract

**Background:**

Cardiac amyloidosis (CA) mimics left ventricular hypertrophy (LVH). It is treatable, but its prognosis is poor. A simple screening tool for CA would be valuable. CA is more precisely diagnosed with echocardiographic deformation parameters (e.g., relative apical sparing pattern [RASP]) than with conventional parameters. We aimed to 1) investigate incremental benefits of echocardiographic deformation parameters over established parameters for CA screening; 2) determine the resultant risk score for CA screening; and 3) externally validate the score in LVH patients.

**Methods:**

We retrospectively studied 295 consecutive non-ischemic LVH patients who underwent detailed diagnostic tests. CA was diagnosed with biopsy or ^99m^Tc-PYP scintigraphy. The base model comprised age (≥65 years [men], ≥70 years [women]), low voltage on the electrocardiogram, and posterior wall thickness ≥ 14 mm in reference to the literature. The incremental benefit of each binarized echocardiographic parameter over the base model was assessed using receiver operating characteristic curve analysis and comparisons of the area under the curve (AUC).

**Results:**

Fifty-four (18%) patients had CA. RASP showed the most incremental benefit for CA screening over the base model. After conducting multiple logistic regression analysis for CA screening using four variables (RASP and base model components), a score was determined (range, 0–4 points). The score demonstrated adequate discrimination ability for CA (AUC = 0.86). This result was confirmed in another validation cohort (178 patients, AUC = 0.88).

**Conclusions:**

We developed a score incorporating RASP for CA screening. This score is potentially useful in the risk stratification and management of LVH patients.

**Supplementary Information:**

The online version contains supplementary material available at 10.1186/s12947-021-00258-x.

## Introduction

Cardiac amyloidosis (CA) is an infiltrative disease of insoluble amyloid proteins in the myocardium. Acquired monoclonal immunoglobulin light-chain (AL) and transthyretin (TTR)-related diseases are the most frequent CA causes. Owing to these infiltrations, ventricular wall thickness and stiffness progress, and thereby CA mimics left ventricular hypertrophy (LVH). Thus, CA can be underdiagnosed in LVH patients [1]. Although CA has a poorer prognosis than other diseases with LVH, CA is currently pharmacologically treatable [2]. A definitive CA diagnosis requires proof of amyloid depositions in cardiomyocytes by endomyocardial biopsy, which may have fatal complications [3]. Bone scintigraphy, including technetium pyrophosphate (^99m^Tc-PYP) scintigraphy, and cardiac magnetic resonance imaging (CMR) are useful for the non-invasive diagnosis of CA; however, they are costly and not available at all facilities [1]. Therefore, CA should be appropriately screened in LVH patients to determine those requiring further work-up.

Symptoms and physical findings are fundamental during CA screening. However, they require specialization, and their interobserver variations are large. Biomarkers, such as B-type natriuretic peptide (BNP) or troponin, are reproducible, widely used, and sensitive, but they are limited by their low specificity for CA [4, 5]. Electrocardiogram (ECG) and echocardiography are real-time diagnostic tools for providing differential diagnosis. Deformation parameters, including the relative apical sparing patterns of longitudinal strain (RASP), help diagnose CA with better precision than conventional parameters [6]. Accordingly, we hypothesized that the inclusion of deformation parameters into established diagnostic parameters would create a risk score for CA screening in LVH patients. We aimed to 1) investigate incremental benefits of echocardiographic parameters, including deformation parameters, over conventional diagnostic parameters for CA screening in patients with LVH; 2) determine the risk score for CA screening using all these variables; and 3) externally validate the score.

## Methods

### Study population

We retrospectively studied 323 consecutive LVH patients who underwent echocardiography and detailed work-up (biopsy, ^99m^Tc-PYP scintigraphy, or CMR) in Ehime University Hospital or Uwajima City Hospital during June 2006–2019. LVH was defined as mean left ventricular (LV) wall thickness > 10 mm for men and > 9 mm for women on echocardiography [7]. We excluded patients with ischemic heart disease and severe aortic stenosis patients; thereafter, 295 patients were enrolled in the final analysis. This study was conducted according to the Declaration of Helsinki and approved by the ethics committee of Ehime University Graduate School of Medicine (IRB: 1905015); the informed consent process used the opt-out method on our hospital websites.

### Clinical and electrocardiographic data

Clinical and electrocardiographic data at the closest time to echocardiography were collected by reviewing the medical chart (**Supplemental Method** **1****and 2**).

### Standard transthoracic echocardiography

Conventional echocardiographic parameters and the parameters that are relatively specific to CA were measured, based on the recommendations of the American Society of Echocardiography and several references (**Supplemental Method 3**).

### Strain imaging

The global longitudinal strain (GLS), ejection fraction strain ratio, and left atrial (LA) strain were measured using offline speckle-tracking analysis (**Supplemental Method 4**).

### Rasp

Quantitatively assessed RASP (qRASP) was calculated by the previously reported formula: qRASP = [average apical LS]/[average basal LS + average mid LS] [6]. qRASP is consistent but requires offline calculation. Some concerns remain regarding the following: 1) dependency on the midventricular strain value, 2) offset of variation of the strain value based on the use of average values, 3) false positive results due to increased strain value of the entire left ventricle, and 4) no established threshold for assessment. Owing to the potential limitation of qRASP assessment, we recently introduced semi-quantitative method of RASP (sRASP) [8, 9]. Currently, GE and Philipps have adopted a color range divided into eight equal parts from − 20% (red) to 20% (blue) when the strain value is represented on a bull’s eye plot. sRASP was defined as reduction in LS of > − 10% in ≥ 5 (of 6) basal segments relative to LS of <− 15% in at least one apical segment.

### Outcomes

The primary outcome was CA diagnosis by biopsy or ^99m^Tc-PYP scintigraphy. Histological CA diagnosis was defined by positive Congo red staining with typical apple green birefringence in each specimen. In most histologically diagnosed CA patients, distinction between AL and TTR-associated amyloidosis (AL-CA and TTR-CA) was performed based on genotyping and/or immunohistochemistry. Patients with CA who showed amyloid infiltration by extra-cardiac biopsy had the diagnosis confirmed by ruling out other causes of LVH using clinical data, echocardiography, CMR, or ^99m^Tc-PYP scintigraphy. Additionally, ^99m^Tc-PYP scintigraphy is relatively specific for TTR-CA imaging [10]. Accordingly, ^99m^Tc-PYP scintigraphy was scored using the following grading system: grade 0, no cardiac uptake; grade 1, mild uptake less than bone; grade 2, moderate uptake equal to bone; and grade 3, high uptake greater than bone [11]. Essentially, the non-invasive diagnosis of TTR-CA using 99mTc-PYP scintigraphy also requires a monoclonal protein assay [10], but since it was not available to all patients in this retrospective study, we expediently defined TTR-CA as cases measuring ≥2 on this score using 99mTc-PYP scintigraphy.

The secondary outcomes were all-cause death and admission for unexpected heart failure after the index echocardiographic examination. Medical records were used to conduct follow-up assessments. Patients were censored at the time of the outcome or at the end of follow-up (December 31, 2019).

### Base model parameters for CA screening

From the aspect of external validity, age (≥65 [men], ≥70 [women]), low voltage on ECG, and LV posterior wall thickness (PWT) ≥14 mm were selected as conventional parameters comprising the base model, referring to previous reports on CA screening models [4, 12–14]. LV wall thickness is a fundamental characteristic of CA [4, 12, 13]. One study demonstrated that PWT was a more useful parameter than interventricular septal wall thickness; therefore, we used PWT [4]. Physical findings and biomarkers were not adopted as model candidates owing to the necessity of expertise and subunit variety.

### Validation

A separate validation group of LVH patients undergoing detailed diagnostic tests (*n* = 242) between June 2006–2019 was obtained from the other three centers (Kitaishikai Hospital, Ehime Prefectural Central Hospital, and Ehime Prefectural Imabari Hospital). Based on the same exclusion criteria with the original cohort, 178 LVH patients were included.

### Statistical analysis

Overall, < 5% of data in the derivation and validation cohorts were missing from patients’ records, except for BNP (8%), NT-proBNP (97%), troponin I (60%), troponin T (97%), serum albumin (6%), HbA1c (9%), total cholesterol (13%), PQ duration (8%), LA reservoir strain (11%), and LA booster strain (25%). Inter- and intraobserver variability of CA specific echocardiographic parameters was assessed using the kappa statistic and intraclass correlation coefficients. Measurements were performed in 30 randomly selected patients by one blinded sonographer and then repeated on more than 14 separate days by two blinded sonographers at Kitaishikai Hospital. The two readers used the same designated movies for assessing consistency. In the strain analysis, the variability included placing the region of interest in the automatically determined cardiac cycle by using a software program. In these 30 selected patients, the assessment time for sRASP and qRASP after constructing a bull’s eye plot for the evaluation of GLS were also measured by the two readers and then averaged.

Categorical variables were expressed as number of events and percentage. Continuous data were expressed as median and interquartile range and compared using Mann-Whitney U test, and categorical variables were compared using χ^2^ or Fisher’s exact tests.

Continuous echocardiographic variables were binarized with external cutoff points to avoid the best clinical scenario and construct a simple, general-purpose, easily implemented scoring system. Cut-off points of each parameter were as follows: PWT ≥14 mm [12], LV ejection fraction ≤55% [5], E/e’ > 12 [5], LA volume index ≥47 mL/m^2^ [15], anterior mitral valve leaflet thickness ≥ 5 mm [16], interatrial septal thickness ≥ 4 mm [17], right ventricular wall thickness ≥ 6 mm [18], GLS ≥ -16% [19], GLS ≥ -17% [5], ejection fraction strain ratio ≥ 3.9% [5], LA reservoir strain < 19% [17], qRASP > 0.87 [15], qRASP > 0.90 [5], and qRASP > 1.00 [6].

A receiver operating characteristic curve (ROC) was used to compare discriminative abilities between the base model and base model plus each echocardiographic parameter for identifying CA. The discrimination ability of each model was estimated as the area under the curve (AUC) using the probability model calculated from multivariable logistic regression for identifying CA. A comparison of AUCs was performed using methods by Delong et al. [20]. The sensitivity and specificity at the maximal Youden index were measured.

The score parameters comprised four parameters, including three base model parameters and the categorical echocardiographic parameter with maximum discriminatory power. Multivariable logistic regression analysis was performed to assess associations between CA diagnosis and the score parameters. The parameter with the lowest regression coefficient among these four variables in the multivariable logistic model was assigned a numeric value of 1, and the other three variables were assigned scores based on values of their regression coefficients relative to those of the lowest value and rounded to the nearest integer. The score was derived by summing the assigned numeric scores. The developed score was validated in the validation sample. Additionally, discrimination ability of the developed score was compared with that of the conventional Rahman’s model comprising interventricular septal thickness > 1.98 cm and low voltage on ECG [13], because subjects, design, and outcome of their study were relatively similar to those of our study. Moreover, to validate the CA screening score more rigorously, discriminative ability of the score was evaluated in selected patients (i.e., biopsy-based patients and patients without atrial fibrillation). Furthermore, discrimination ability of the score for identifying CA subtypes (AL-CA and TTR-CA) was assessed in all enrolled patients.

Additionally, the association of the score with adverse events was assessed using univariable Cox proportional hazards models and Kaplan-Meier curves. No significant violations of assumption of proportional hazards were noted. Differences in survival between groups were assessed using the log-rank test.

Threshold significance was defined as *p* < 0.05. Statistical analysis was performed using the R statistical package ver. 3.5.3 (R Foundation for Statistical Computing, Vienna, Australia, available online at http://www.R-project.org).

## Results

### Outcomes and patient characteristics

Of the 295 LVH patients, 54 were diagnosed with CA. Among these, 48 and 6 patients had biopsy-proven and ^99m^Tc-PYP scintigraphy-proven amyloidosis, respectively. Biopsies were obtained from the myocardium in 38 patients. Twenty-two (41%) and 23 (43%) patients were diagnosed with AL-CA and TTR-CA, respectively. CA type could not be identified in nine patients (17%) who were diagnosed before 2010. They were older and frail; therefore, there was no indication for active treatment at the time, and their CA type was not investigated. The etiology of LVH in the remaining 241 patients was hypertrophic cardiomyopathy (*n* = 120), hypertensive heart disease (*n* = 74), dilated cardiomyopathy (*n* = 16), cardiac sarcoidosis (*n* = 14), valvular heart disease (*n* = 12), LV non-compaction cardiomyopathy (*n* = 2), Fabry disease (*n* = 2), and mitochondrial cardiomyopathy (*n* = 1).

Table 1 summarizes the baseline clinical and echocardiographic parameters of the enrolled patients. CA patients were significantly older and had lower voltage, thicker LV wall, more deteriorated LV diastolic functional and strain imaging parameters, and higher frequency of RASP than those without CA.

**Table 1:** Baseline characteristics in patients with and without cardiac amyloidosis

Variables	Available data	Overall (*n* = 295)	CA (*n* = 54)	Non-CA (*n* = 241)	***p*** value (CA vs Non-CA)
Age (years)	295	67 (55–75)	75 (68–79)	65 (55–72)	**< 0.01**
Male sex, n (%)	295	191 (65)	47 (87)	144 (60)	**< 0.01**
Body weight (kg)	295	61.9 (53.0–72.0)	57.5 (53.0–65.8)	62.0 (53.0–74.0)	**0.048**
Body mass index (kg/m^2^)	295	23.6 (21.1–26.7)	22.1 (20.6–24.5)	24.0 (21.4–27.1)	**< 0.01**
Systolic blood pressure (mmHg)	294	129 (112–146)	111 (100–125)	133 (117–150)	**< 0.01**
Diastolic blood pressure (mmHg)	294	71 (62–80)	63 (56–72)	72 (64–82)	**< 0.01**
Heart rate (/min)	295	69 (60–79)	72 (66–78)	68 (59–79)	0.08
NYHA functional class at discharge (I/II/III/IV), n (%)	295	149/59/49/38 (50/20/17/13)	13/18/16/7 (24/33/30/13)	136/41/33/31 (56/17/14/13)	**< 0.01**
**Comorbidities**					
Hypertension, n (%)	295	141 (48)	16 (30)	125 (52)	**< 0.01**
Diabetes, n (%)	295	57 (19)	4 (7)	53 (22)	**0.01**
Dyslipidemia, n (%)	295	85 (29)	12 (22)	73 (30)	0.32
Atrial fibrillation, n (%)	295	74 (25)	14 (26)	23 (10)	**< 0.01**
Device (N/PPM/ICD/CRT), n (%)	295	263/16/11/5 (89/5/4/2)	43/7/1/3 (80/13/2/6)	220/9/10/2 (91/4/4/1)	**< 0.01**
**Serum markers**					
B-type natriuretic peptide (pg/mL)	272	123.9 (49.8–352.3)	263.7 (135.2–860.3)	109.5 (44.9–271.1)	**< 0.01**
Troponin positive^*^, n (%)	79	51 (65)	19 (86)	32 (56)	**0.01**
Hemoglobin (g/L)	290	13.3 (11.9–14.8)	13.0 (11.5–14.3)	13.5 (11.9–15.0)	0.07
eGFR (mL/min/1.73 m^2^)	292	61.1 (46.1–73.6)	52.3 (40.5–67.9)	62.0 (48.8–73.9)	**0.01**
Sodium (mmol/L)	292	140 (138–142)	139 (136–141)	140 (139–142)	**< 0.01**
Serum albumin (mg/L)	287	4.0 (3.5–4.3)	3.7 (3.1–4.0)	4.1 (3.7–4.3)	**< 0.01**
**Electrocardiographic variables**					
SV_1_ + RV_5_ voltage (mm)	295	3.1 (1.9–4.1)	1.8 (1.3–2.4)	3.3 (2.2–4.4)	**< 0.01**
PQ duration (ms)	255	174 (157–202)	195 (160–239)	172 (156–197)	**0.02**
QRS duration (ms)	295	102 (92–116)	108 (95–138)	102 (92–114)	**0.01**
Heart-rate-corrected QT (ms)	295	438 (418–459)	448 (423–467)	437 (416–453)	**< 0.01**
Right bundle branch block, n (%)	295	27 (9)	8 (15)	19 (8)	0.12
Left bundle branch block, n (%)	295	18 (6)	7 (13)	11 (5)	**0.03**
Pseudo-infarct pattern, n (%)	295	48 (16)	16 (30)	32 (13)	**< 0.01**
Low voltage, n (%)	295	11 (3)	7 (13)	4 (2)	**< 0.01**
**Conventional echocardiographic variables**					
Interventricular septal thickness (mm)	295	13.0 (11.0–15.0)	14.0 (13.0–15.6)	12.0 (11.0–15.0)	**< 0.01**
LV posterior wall thickness (mm)	295	11.0 (10.0–13.0)	13.0 (11.0–15.0)	11.0 (10.0–12.0)	**< 0.01**
LV mean wall thickness (mm)	295	11.9 (10.5–13.5)	13.5 (11.6–15.4)	11.5 (10.5–13.0)	**< 0.01**
LV mass index (g/m^2^)	295	137.5 (112.5–169.3)	140.2 (114.0–179.1)	136.5 (112.0–168.6)	0.35
LV end-diastolic diameter (mm)	295	48.0 (43.0–54.0)	45.0 (40.0–49.0)	49.0 (44.0–54.0)	**< 0.01**
LV end-systolic diameter (mm)	295	31.0 (25.6–37.0)	31.0 (27.0–35.0)	31.0 (25.0–39.0)	0.59
LV end-diastolic volume (mL)	295	75.0 (59.1–101.0)	72.0 (59.2–88.8)	76.0 (59.1–105.7)	0.19
LV end-systolic volume (mL)	295	30.0 (21.0–46.5)	32.3 (22.3–40.3)	29.0 (21.0–48.5)	0.84
LV ejection fraction (%)	295	59.3 (48.8–66.7)	55.1 (49.9–62.9)	61.0 (48.5–66.9)	0.16
E velocity deceleration time (ms)	295	200 (161–245)	169 (145–200)	208 (168–256)	**< 0.01**
e’-wave velocity (cm/s)	289	4.3 (3.4–5.6)	4.0 (2.8–4.5)	4.6 (3.6–5.8)	**< 0.01**
Septal E/e’	289	15.2 (11.5–20.5)	19.6 (16.2–26.6)	14.0 (11.1–18.3)	**< 0.01**
LA volume index (mL/m^2^)	292	42.7 (30.0–54.6)	48.7 (40.2–60.9)	40.7 (29.1–53.4)	**< 0.01**
Moderate to severe mitral regurgitation, n (%)	293	40 (14)	11 (20)	29 (12)	0.13
Moderate to severe aortic regurgitation, n (%)	293	19 (6)	5 (9)	14 (6)	0.36
Pericardial effusion, n (%)	286	22 (8)	6 (11)	16 (7)	0.26
Granular sparkling, n (%)	285	38 (13)	10 (19)	28 (12)	0.18
Interatrial septal wall thickness (mm)	282	6.7 (5.5–7.9)	6.6 (4.9–8.7)	6.8 (5.5–7.8)	0.55
Right ventricular wall thickness (mm)	285	3.8 (3.2–4.4)	3.9 (3.4–4.4)	3.7 (3.2–4.3)	0.20
Anterior mitral valve leaflet thickness (mm)	285	2.9 (2.4–3.5)	3.2 (2.7–3.5)	2.9 (2.4–3.5)	0.12
**Strain imaging variables**					
LV global longitudinal strain (%)	287	−11.8 (−15.1–-8.6)	−10.0 (−12.5–-7.5)	−12.4 (−15.3–-8.7)	**< 0.01**
Ejection fraction strain ratio	287	4.8 (3.9–6.1)	5.4 (4.4–6.8)	4.6 (3.8–6.0)	**0.01**
LA longitudinal strain (reservoir phase) (%)	260	14.9 (9.1–22.2)	7.7 (6.1–12.0)	17.5 (12.3–23.8)	**< 0.01**
LA longitudinal strain (booster phase) (%)	226	8.7 (5.0–11.8)	3.5 (1.9–5.2)	9.6 (6.4–12.8)	**< 0.01**
qRASP	287	0.69 (0.50–0.84)	1.02 (0.84–1.19)	0.63 (0.47–0.77)	**< 0.01**
qRASP > 1.00, n (%)	287	41 (14)	29 (55)	12 (5)	**< 0.01**
qRASP > 0.90, n (%)	287	55 (19)	36 (68)	19 (8)	**< 0.01**
qRASP > 0.87, n (%)	287	62 (22)	37 (70)	25 (11)	**< 0.01**
sRASP, n (%)	287	42 (15)	26 (49)	15 (6)	**< 0.01**

Data are expressed as the median (interquartile range), or number (percentage)

^*^Troponin I (*n* = 68) ≥26.2 pg/mL or Troponin T (*n* = 11) ≥0.014 ng/mL

ACEi indicates angiotensin-converting-enzyme inhibitor; AF, atrial fibrillation; ARB, angiotensin receptor blocker; CA, cardiac amyloidosis; CRT, cardiac resynchronization therapy; ICD, implantable cardioverter-defibrillator; LA, left atrial; LV, left ventricular; NYHA, New York Heart Association; PPM, permanent pacemaker; qRASP, quantitatively assessed relative apical sparing pattern of longitudinal strain; sRASP, semi-quantitatively assessed relative apical sparing pattern of longitudinal strain

### Incremental benefits of echocardiographic parameters over the base model

The discriminative ability of the base screening model comprising age (≥65 [men], ≥70 [women]), low voltage on ECG, and PWT ≥14 mm for identifying CA was acceptable. Of the binarized echocardiographic parameters, only RASP showed an incremental benefit over the base model **(**Table 2**)**. Additionally, we inspected the additive value of the continuous variables over the base model to confirm whether the cutoff value used for each echo parameter was appropriate. Of the continuous echocardiographic parameters, adding qRASP resulted in the largest discriminatory power **(Supplemental Table** **1****)**.

**Table 2:** Incremental benefits of categorical echocardiographic parameters over the base model

	AUC (95% CI)	p value (base model vs base model plus each echo parameter)
**Base model**: Age (≥65 [men], ≥70 [women]) + Low voltage+ PWT ≥14.0 mm	0.82 (0.76–0.88)	
+ LV ejection fraction ≤55%	0.83 (0.76–0.90)	0.67
+ E/e’ > 12	0.84 (0.78–0.90)	0.34
+ LA volume index ≥47 (mL/m^2^)	0.84 (0.77–0.91)	0.13
+ Pericardial effusion	0.83 (0.76–0.89)	0.37
+ Granular sparkling	0.82 (0.75–0.88)	0.82
+ Interatrial septal thickness ≥ 4 mm	0.83 (0.77–0.89)	0.54
+ Right ventricular wall thickness ≥ 6 mm	0.81 (0.75–0.88)	0.58
+ Anterior mitral valve leaflet thickness ≥ 5 mm	0.81 (0.74–0.87)	0.49
+ GLS ≥ -16%	0.82 (0.75–0.88)	0.98
+ GLS ≥ -17%	0.82 (0.75–0.88)	0.90
+ Ejection fraction strain ratio > 3.9%	0.83 (0.77–0.90)	0.13
+ LA reservoir strain < 19%	0.84 (0.78–0.90)	0.16
+ qRASP > 1.00	0.87 (0.80–0.93)	**< 0.01**
+ qRASP > 0.90	0.87 (0.80–0.93)	**< 0.01**
+ qRASP > 0.87	0.86 (0.80–0.93)	**0.01**
+ sRASP	0.87 (0.81–0.93)	**0.03**

AUC indicates area under the curve; CI, confidence interval; GLS, global longitudinal strain; LA, left atrial; LV, left ventricular; PWT, posterior wall thickness; qRASP, quantitatively assessed relative apical sparing pattern of longitudinal strain; sRASP, semi-quantitatively assessed relative apical sparing pattern of longitudinal strain

### Development of the CA screening score

**Fig. 1:**
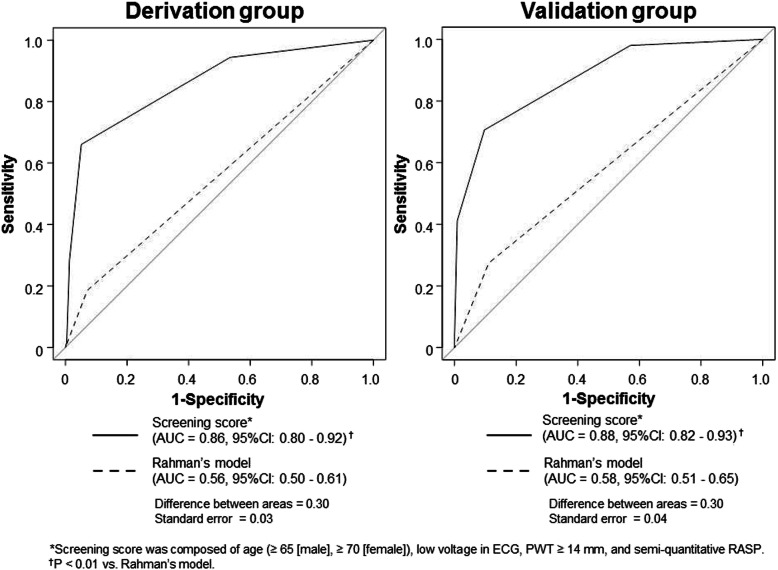
Comparison of the screening score with Rahman’s model in the derivation (left) and validation groups (right). AUC; area under the curve; CI, confidence interval; PWT, posterior wall thickness; RASP, relative apical sparing pattern

**Fig. 2:**
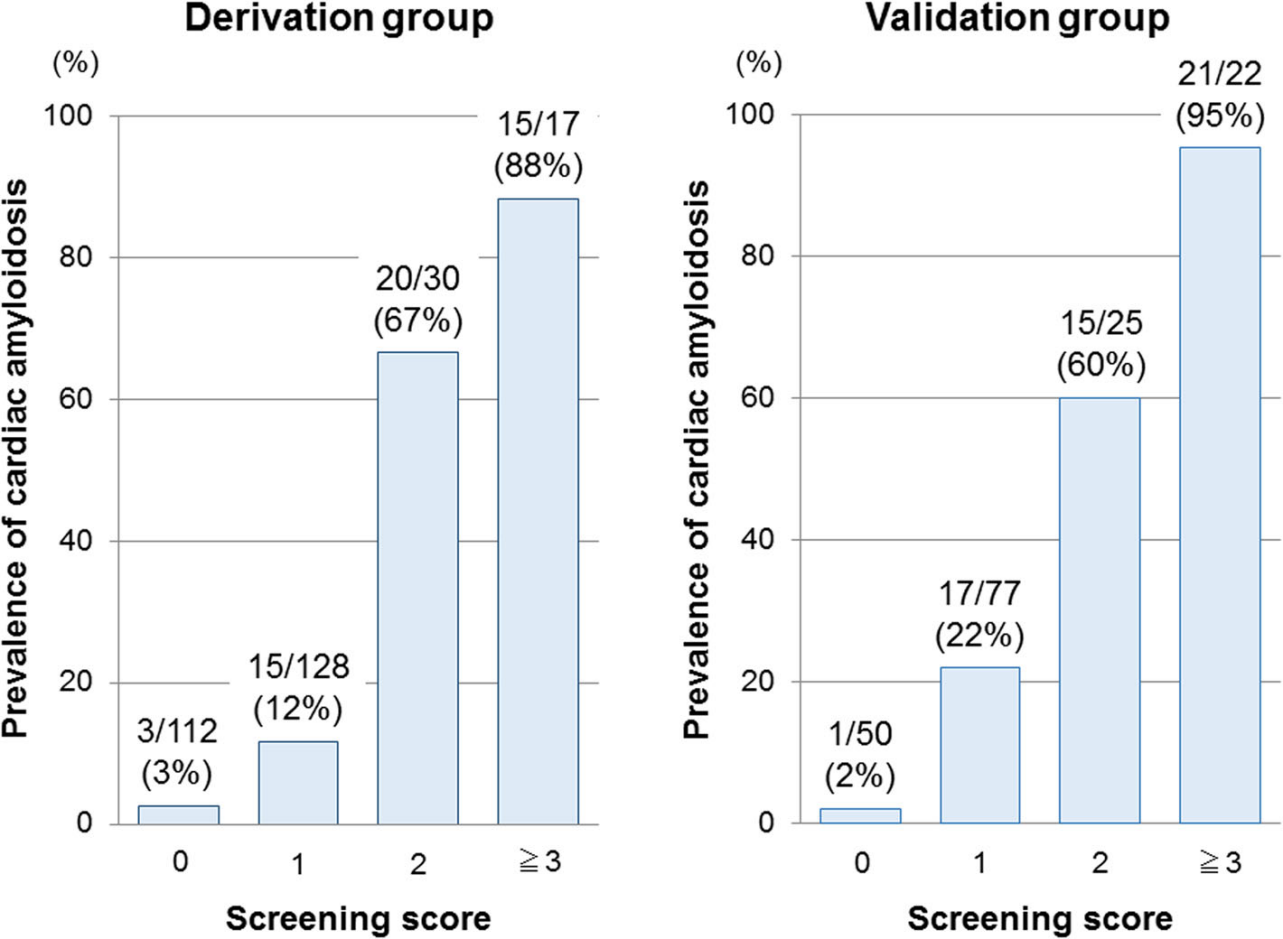
Association between the prevalence of cardiac amyloidosis and the screening score in the derivation (left) and validation groups (right)

Accordingly, we created the diagnostic CA screening model using three base model parameters plus RASP **(**Table 3**)**. We selected sRASP of the categorical RASP parameters because it could be quickly assessed online at the patient’s bedside, and it demonstrated similar discriminatory ability to other categorized qRASPs [18]. All parameters were independently associated with CA. Each parameter was assigned 1 point based on its relative effect. A score was constructed by adding the numeric values of factors identified in each patient, and the score range was 0–4. The mean score was 0.8 ± 0.9. Using the ROC analysis to identify CA, the score showed optimal discriminative ability, significantly better than that of the conventional Rahman’s model **(**Fig. 1**, left)**. A total score of ≥2 showed optimal sensitivity (66%), specificity (95%), positive predictive value (74%), and negative predictive values (92%). The prevalence of CA clearly increased as the sum of the screening score increased **(**Fig. 2**, left).**

**Table 3:** Multivariable logistic regression analysis of the base model parameters and semi-quantitative relative apical strain pattern for identifying cardiac amyloidosis (*n* = 287; cardiac amyloidosis = 53)

Variables	β	OR (95% CI), p value	Score
Age (≥65 [men], ≥70 [women])	2.0	7.4 (3.1–17.7), **< 0.01**	1
Low voltage in ECG	2.4	11.3 (2.3–56.0), **< 0.01**	1
PWT ≥14 mm	1.8	5.8 (2.4–14.2), **< 0.01**	1
sRASP	2.4	11.3 (4.5–28.5), **< 0.01**	1

CI indicates confidence interval; ECG, electrocardiogram; OR, odds ratio; PWT, posterior wall thickness; sRASP, semi-quantitatively assessed relative apical sparing pattern of longitudinal strain

### Validation

Score accuracy was investigated in the validation cohort. Patients’ characteristics were similar to those of the derivation cohort **(****Supplemental Table 2****)**. Of 178 LVH patients, 56 were diagnosed with CA, including 26 and 30 patients with biopsy-proven and ^99m^Tc-PYP scintigraphy-proven amyloidosis, respectively. Eleven patients (20%) were diagnosed with AL-CA and 44 (78%) with TTR-CA. CA type could not be determined in one patient (2%). The mean score of this cohort was 1.1 ± 1.0. Even in the validation cohort, the score showed optimal discriminative ability, significantly better than the Rahman’s model **(**Fig. 1**, right)**. Similarly, a total score of ≥2 showed optimal sensitivity (71%), specificity (93%), positive predictive value (77%), and negative predictive values (85%). Furthermore, the positive relationship between CA prevalence and score was similar to that in the derivation cohort **(**Fig. 2**, right)**.

### Discriminative ability of the score incorporating binarized qRASP instead of sRASP for identifying CA

The measurement method of sRASP has not been fully validated compared to that of qRASP. Therefore, we inspected the discriminative ability of the score incorporating binarized qRASP, instead of sRASP. In this situation, qRASP > 0.90 was chosen because it showed greater additive value over the base model in the derivation cohort, rather than qRASP > 1.00 and > 0.87. qRASP > 0.90 was assigned 1 point and was used as a score component instead of sRASP. The score incorporating qRASP > 0.90 exhibited the same discrimination ability as that incorporating sRASP in both cohorts **(****Supplemental Fig. 1****)**.

### Discriminative ability of the score for identifying CA in selected patients

Analyses in selected patients with a definitive histological diagnosis or in patients without atrial fibrillation, who usually provide more accurate echo results, may contribute to validation of the present results. We also investigated the discriminative ability of the score in biopsy-proven patients (*n* = 204) and patients without atrial fibrillation (*n* = 336) who were successfully assessed using the score. Of these selected patients, CA was diagnosed in 74 and 68 patients, respectively. Even in these patients, the AUC of the score was almost equivalent to that in all enrolled patients and significantly better than that of the Rahman’s model **(****Supplemental Fig. 2****)**.

### Discriminative ability of the score for identifying CA subtypes

**Fig. 3:**
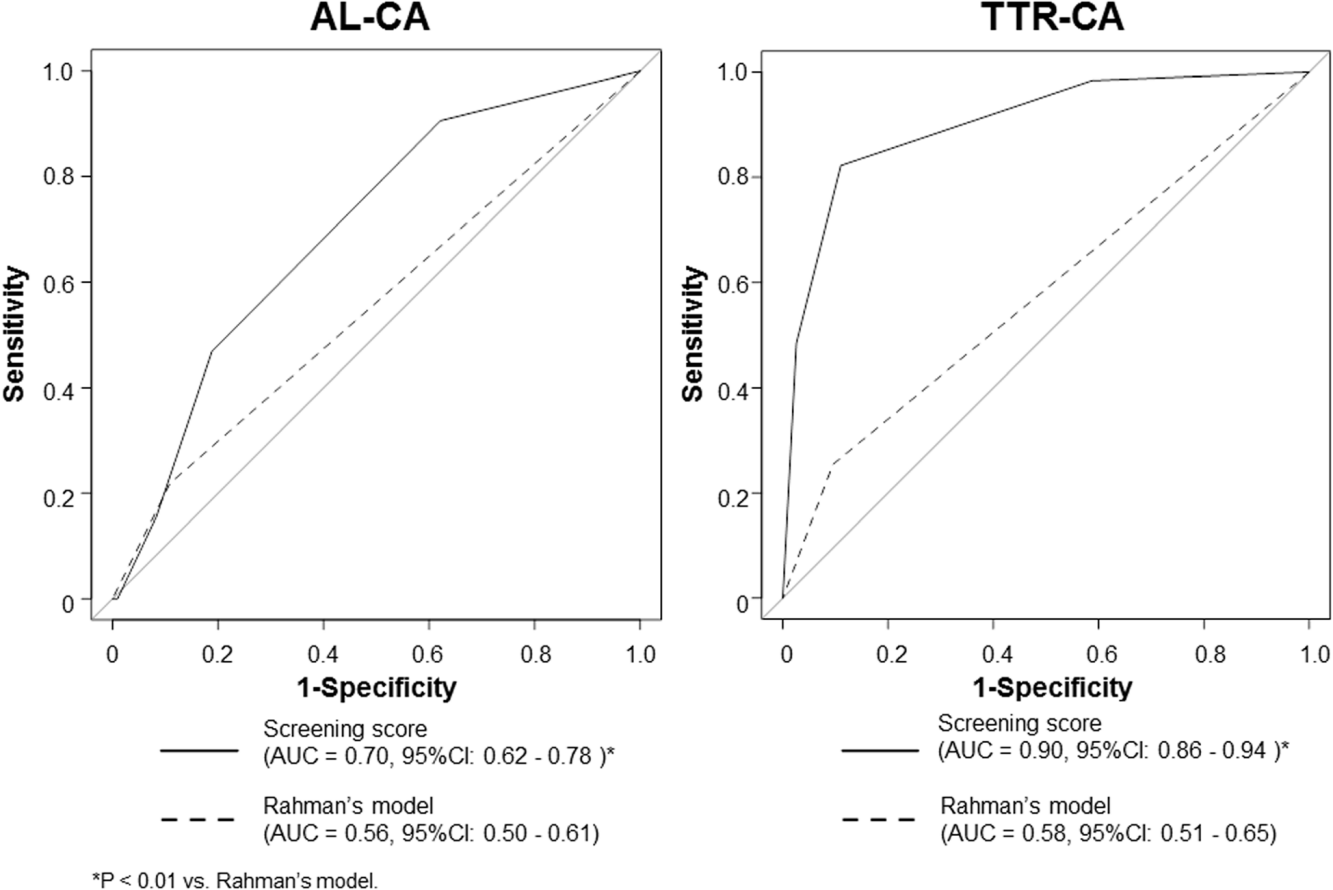
Comparison of the screening score with Rahman’s model for identifying cardiac amyloidosis subtypes in enrolled patients. AUC; area under the curve; AL-CA, light-chain types of cardiac amyloidosis; CI, confidence interval; PWT, posterior wall thickness; RASP, relative apical sparing pattern; TTR-CA, transthyretin types of cardiac amyloidosis

The histological feature of cardiac involvement in TTR-CA and AL-CA is different [21]. In all enrolled patients with successful score assessment (*n* = 461), we performed ROC analysis to assess the discriminative ability of the score for identifying CA subtypes. The score discriminated AL-CA (*n* = 33) more accurately than Rahman’s model, but its discriminative ability was modest **(**Fig. 3**, left)**. For this discrimination, a total score of ≥2 showed optimal sensitivity (46%) and specificity (82%). However, the score still accurately discriminated TTR-CA (*n* = 67) **(**Fig. 3**, right)**. For this discrimination, a total score of ≥2 showed optimal sensitivity (80%) and specificity (90%).

### Predictive ability of adverse events with the score

**Fig. 4:**
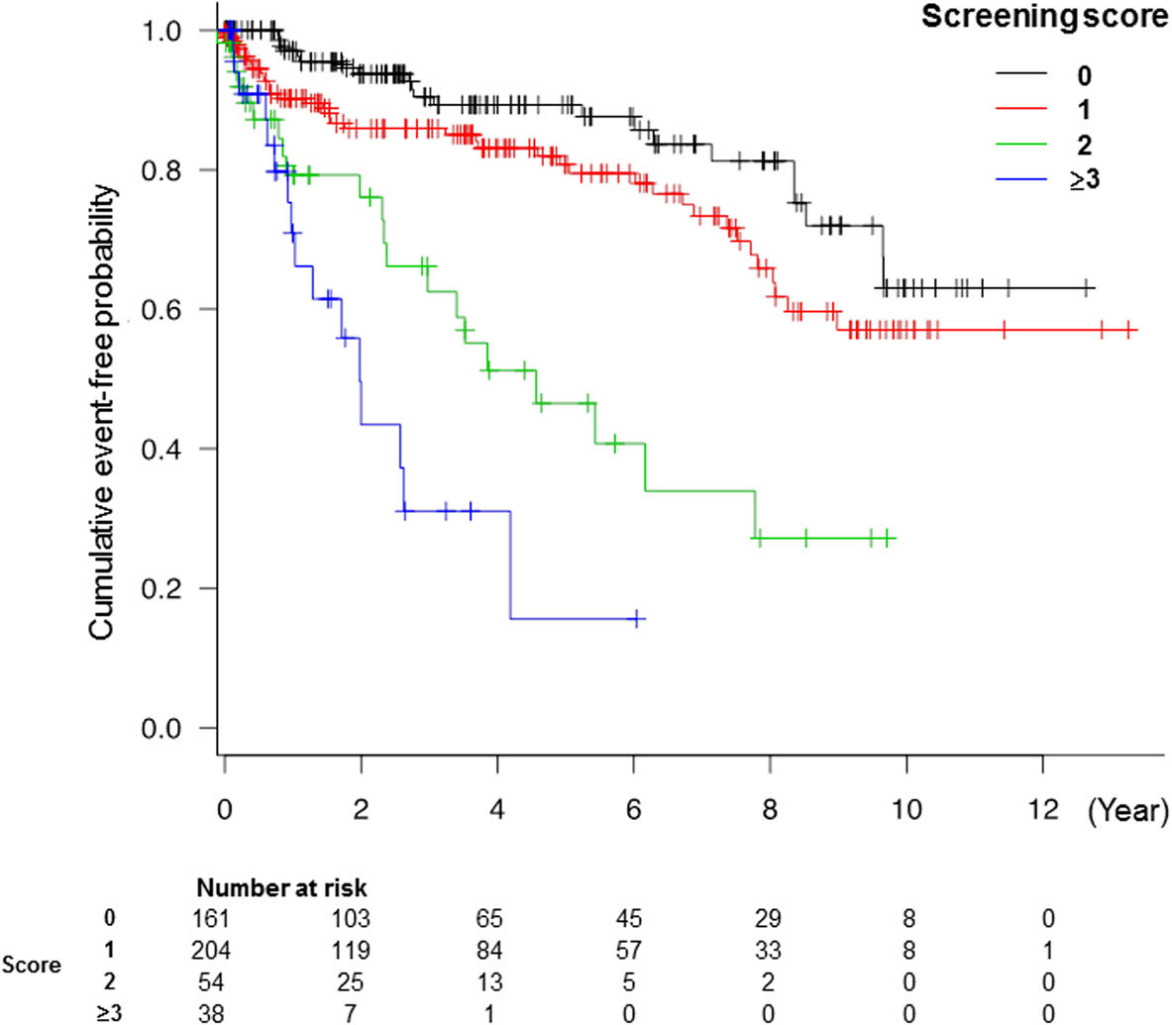
Kaplan-Meier estimates of time to the occurrence of adverse events according to the screening score

Of patients with the score and follow-up data (*n* = 456; median follow-up: 2.6 years, IQR: 0.8–5.8 years), 27 (6%) suffered all-cause death, 79 (17%) presented with admission for unexpected heart failure, and 106 (23%) experienced both. The score was significantly associated with the adverse outcome (hazard ratio, 2.12; 95% confidence interval, 1.74–2.59; *p* < 0.01). In the Kaplan-Meier curves, the incidence of adverse outcomes significantly increased as the score increased (log-rank test, *p* < 0.01) **(**Fig. 4**)**.

### Reproducibility

Reproducibility data are summarized in **Supplemental Table 3**. GLS, qRASP, and sRASP demonstrated excellent consistency. After constructing a bull’s eye plot for the evaluation of GLS, the averaged assessment times for sRASP were significantly shorter than those for qRASP (1 ± 2 s vs. 63 ± 6 s, *p* < 0.01).

## Discussion

In this study, we investigated the incremental benefits of echocardiographic deformation parameters versus established parameters for CA screening, determined the resultant risk score for CA screening, and externally validated the score in LVH patients. We developed a risk score, comprising four parameters (age, low voltage in electrocardiography, PWT ≥14 mm, and RASP), which has potential utility in the risk stratification and management of LVH patients.

### Strength of the present CA screening score

According to an expert consensus, the first screening tests in suspected CA patients are symptoms, ECG, echocardiography, CMR, and biomarkers [22]. However, symptoms are highly subjective, and CMR is relatively expensive and not widely accessible. Biomarkers are reproducible, widely used and sensitive, but their limitation is a low specificity for CA. Conversely, ECG and echocardiography are commonly used in various clinical settings, and their parameters are highly reproducible. Therefore, referring to previous consensus reports describing CA screening models, we selected the three indices (age, low voltage, and PWT) with high reproducibility and versatility as basic model parameters for CA screening [4, 12–14]. The high discriminatory power of the base model (AUC in the derivation cohort: 0.82) may indicate that the parameter selection was relatively appropriate.

Several strain indices (RASP, ejection fraction strain ratio, and LA strain) are more useful in CA screening than conventional indices [6, 15, 16]. Here, the incremental benefit of several binary echocardiographic indicators over the base model in CA identification was analyzed. RASP showed the most additional value, and the result was the same even using continuous variables. Accordingly, our CA screening score comprised base model parameters and RASP. The discrimination ability of the score was significantly better than that of the conventional Rahman’s model, and it was well-validated even in the selected cases, such as biopsied patients and patients without atrial fibrillation. This finding may indicate that the score would be highly versatile in clinical practice. Additionally, an increased score was significantly associated with a poorer prognosis, likely reflected by the fact that CA has a poorer prognosis than other hypertrophic diseases [23]. This result may demonstrate the validity of the prediction accuracy of the present score.

Recently, Boldrini et al. reported the multiparametric echocardiographic score for the diagnosis of CA in patients with LVH in an international cohort study [24]. Their score adopted longitudinal strain and systolic apex-to-base ratio similar to RASP, which demonstrated an excellent CA discrimination ability; this might support the usefulness of our scores using RASP. We adopted semi-quantitatively assessed RASP to the score. This parameter has high reproducibility, does not require offline analysis, and can be evaluated simultaneously with GLS measurement [8]. This would make the present score practical in clinical settings. Nonetheless, sRASP has not been fully validated yet. However, even when the binary RASP obtained using the conventional quantitative method was incorporated into the score instead of sRASP, the discrimination ability of the score was similar to the score incorporating sRASP. Thus, it may be feasible to adapt the binary variable of qRASP as a component of the score, instead of sRASP, especially when evaluating RASP on non-General Electronic machines.

### Differences in discriminative abilities for identifying CA subtypes

Although AL-CA and TTR-CA have different pathological conditions, it is difficult to distinguish these etiologies at an early stage in clinical practice [21]. Therefore, in the present study, we created a score to screen for both CA phenotypes to differentiate them from other causes of LV hypertrophy. Consequently, the present score was more suitable for TTR-CA than for AL-CA screening. There are a few possible reasons for this result. First, the model components of the score may have an impact. AL-CA often is diagnosed at a younger age than TTR-CA [1]. Moreover, the wall thickness of AL-CA is generally thinner than that of TTR-CA [1]. Conversely, low voltage on electrocardiogram is more common in AL than in TTR, but the incidence of RASP seems similar between AL and TTR [1, 25]. Therefore, the preference of each model component may have been more advantageous in screening TTR-CA than AL-CA. Second, this result may be involved in the enrollment of patients with already increased LV wall thickness. Therefore, some AL-CA patients who generally have thinner walls than those with TTR-CA [1] were not included, which may have led to selection bias.

### Limitations

Our data should be interpreted while considering the limitations. First, biopsy data were not available for all cases. However, all patients were diagnosed with a detailed work up by cardiology specialists. Also, the accuracy of the scoring system was consistent between overall and biopsied patients. Second, a monoclonal protein assay was not available to all patients. Therefore, in our sample, TTR-CA patients diagnosed by ^99m^Tc-PYP scintigraphy might overlap with other diseases, especially AL-CA. However, the purpose of our score is to screen for all types of CA, and the discrimination ability of the score in sub-group analysis of only biopsy-proven cases was almost equivalent to that in all enrolled patients. Third, some laboratory data useful for CA screening (troponin T, NT-proBNP, and serum kappa/lambda free light chain ratio) were unavailable because the measurement facilities were limited. Consequently, the accuracy of the present score could not be compared with models using these laboratory data [4, 5]. Furthermore, the present score was more suitable for TTR-CA than AL-CA. Therefore, in order to identify AL-CA, it may be important to use the AL score by Boldrini et al. and serum free light chain assay in combination with our score [24]. Forth, RASP was difficult to assess in 11 cases with arrhythmia or poor echocardiographic imaging (2%). However, a prospective assessment may reduce this rate. Fifth, strain analysis is not always available at all facilities. Recently, Aimo et al. proposed a CA screening score that uses only relative wall thickness and E/e’ without strain analysis, which could be an alternative method in this situation [26]. Sixth, all echocardiographic measurements were calculated as the average value in three cardiac cycles. However, the measurements should be averaged in five cycles, for example, when there was atrial fibrillation. Seventh, the target of this study was patients with already increased LV wall thickness. Therefore, the validity of the present score in individuals with normal LV thickness is unknown. However, increased LV wall thickness is a major characteristic of CA, and the enrollment method of this study seems mostly relevant to a real clinical setting. Eighth, echocardiographic examinations were performed using three different ultrasound machines. The difference may partially affect the reproducibility of the data. Finally, this study was retrospective in design. The retrospective analysis had limitations with respect to potential confounders and risk for bias. Thus, larger multicenter prospective studies are warranted to confirm our results.

## Conclusion

We developed a CA screening score incorporating RASP, which presents better accuracy than that of the conventional prediction model. This score can identify patients who require subsequent work-up, including biopsy and scintigraphy, and consequently facilitate early pharmacological intervention and improve their prognosis. However, symptoms and biomarkers are fundamental assessment methods to screen CA. The present score should be considered as an additional tool to the biohumoral assessment.

## Supplementary Information

**Additional file 1 Supplemental Method 1:** Clinical data. **Supplemental Method 2:** Electrocardiogram (ECG). **Supplemental Method 3:** Standard transthoracic echocardiography. **Supplemental Method 4:** Strain imaging. **Supplemental Table 1:** Incremental benefits of continuous echocardiographic parameters over the base model. **Supplemental Table 2:** Baseline characteristics of patients with and without cardiac amyloidosis in the validation cohort. **Supplemental Table 3:** Reliability data.

**Additional file 2 Supplemental Fig. 1:** Comparison between the screening score based on base model parameters and semi-quantitatively assessed RASP and based on base model parameters and quantitatively assessed RASP > 0.90 in the derivation (left) and validation groups (right). AUC; area under the curve; CI, confidence interval; qRASP, quantitative relative apical sparing pattern; sRASP, semi-quantitative relative apical sparing pattern.

**Additional file 3 Supplemental Fig. 2:** Comparison of the new screening score with Rahman’s model in the selected patients. AUC; area under the curve; CA, cardiac amyloidosis; CI, confidence interval; PWT, posterior wall thickness; RASP, relative apical sparing pattern.

## Data Availability

The datasets used and/or analyzed during the current study are available from the corresponding author on reasonable request.

## Abbreviations

AL: amyloid light-chain
AL-CA: amyloid light-chain-associated cardiac amyloidosis
AUC: area under the curve
BNP: B-type natriuretic peptide
CA: cardiac amyloidosis
CMR: cardiac magnetic resonance imaging
e’: early diastolic mitral annular velocity
ECG: electrocardiogram
GLS: global longitudinal strain
LA: left atrial
LS: longitudinal strain
LV: left ventricular
LVH: left ventricular hypertrophy
NT-proBNP: N terminal-pro B-type natriuretic peptide
PWT: posterior wall thickness
RASP: relative apical sparing pattern of longitudinal strain
ROC: receiver operating characteristic curve
qRASP: quantitatively assessed relative apical sparing pattern of longitudinal strain
sRASP: semi-quantitatively assessed relative apical sparing pattern of longitudinal strain
TTR: transthyretin
TTR-CA: transthyretin-associated cardiac amyloidosis
^99m^Tc-PYP: technetium pyrophosphate

## References

[1] Maurer MS, Elliott P, Comenzo R, Semigran M, Rapezzi C (2017). Addressing common questions encountered in the diagnosis and management of cardiac amyloidosis. Circulation..

[2] Maurer MS, Schwartz JH, Gundapaneni B, Elliott PM, Merlini G, Waddington-Cruz M, Kristen AV, Grogan M, Witteles R, Damy T, Drachman BM, Shah SJ, Hanna M, Judge DP, Barsdorf AI, Huber P, Patterson TA, Riley S, Schumacher J, Stewart M, Sultan MB, Rapezzi C (2018). Tafamidis treatment for patients with transthyretin amyloid cardiomyopathy. N Engl J Med.

[3] From AM, Maleszewski JJ, Rihal CS (2011). Current status of endomyocardial biopsy. Mayo Clin Proc.

[4] Marume K, Takashio S, Nishi M, Hirakawa K, Yamamoto M, Hanatani S, Oda S, Utsunomiya D, Shiraishi S, Ueda M, Yamashita T, Sakamoto K, Yamamoto E, Kaikita K, Izumiya Y, Yamashita Y, Ando Y, Tsujita K (2019). Combination of commonly examined parameters is a useful predictor of positive (99 m)Tc-labeled pyrophosphate scintigraphy findings in elderly patients with suspected transthyretin cardiac amyloidosis. Circ J.

[5] Nicol M, Baudet M, Brun S, Harel S, Royer B, Vignon M, Lairez O, Lavergne D, Jaccard A, Attias D, Macron L, Gayat E, Cohen-Solal A, Arnulf B, Logeart D (2020). Diagnostic score of cardiac involvement in AL amyloidosis. Eur Heart J Cardiovasc Imaging.

[6] Phelan D, Collier P, Thavendiranathan P, Popović ZB, Hanna M, Plana JC, Marwick TH, Thomas JD (2012). Relative apical sparing of longitudinal strain using two-dimensional speckle-tracking echocardiography is both sensitive and specific for the diagnosis of cardiac amyloidosis. Heart..

[7] Marwick TH, Gillebert TC, Aurigemma G, Chirinos J, Derumeaux G, Galderisi M, Gottdiener J, Haluska B, Ofili E, Segers P, Senior R, Tapp RJ, Zamorano JL (2015). Recommendations on the use of echocardiography in adult hypertension: a report from the European Association of Cardiovascular Imaging (EACVI) and the American Society of Echocardiography (ASE). J Am Soc Echocardiogr.

[8] Saito M, Imai M, Wake D, Higaki R, Nakao Y, Sumimoto T, Yokomoto Y, Ogimoto A, Suzuki M, Kawakami H, Hiasa G, Okayama H, Inoue K, Ikeda S, Yamaguchi O (2020). Semi-quantitative assessment of the relative apical sparing pattern of longitudinal strain for cardiac amyloidosis identification. Echocardiography..

[9] Saito M, Imai M, Wake D, Higaki R, Nakao Y, Morioka H (2020). Prognostic assessment of relative apical sparing pattern of longitudinal strain for severe aortic valve stenosis. Int J Cardiol Heart Vasc.

[10] Gillmore JD, Maurer MS, Falk RH, Merlini G, Damy T, Dispenzieri A, Wechalekar AD, Berk JL, Quarta CC, Grogan M, Lachmann HJ, Bokhari S, Castano A, Dorbala S, Johnson GB, Glaudemans AWJM, Rezk T, Fontana M, Palladini G, Milani P, Guidalotti PL, Flatman K, Lane T, Vonberg FW, Whelan CJ, Moon JC, Ruberg FL, Miller EJ, Hutt DF, Hazenberg BP, Rapezzi C, Hawkins PN (2016). Nonbiopsy diagnosis of cardiac transthyretin amyloidosis. Circulation..

[11] Perugini E, Guidalotti PL, Salvi F, Cooke RM, Pettinato C, Riva L (2005). Noninvasive etiologic diagnosis of cardiac amyloidosis using 99mTc-3,3-diphosphono-1,2-propanodicarboxylic acid scintigraphy. J Am Coll Cardiol.

[12] Witteles RM, Bokhari S, Damy T, Elliott PM, Falk RH, Fine NM, Gospodinova M, Obici L, Rapezzi C, Garcia-Pavia P (2019). Screening for transthyretin amyloid cardiomyopathy in everyday practice. JACC Heart Fail.

[13] Rahman JE, Helou EF, Gelzer-Bell R, Thompson RE, Kuo C, Rodriguez ER, Hare JM, Baughman KL, Kasper EK (2004). Noninvasive diagnosis of biopsy-proven cardiac amyloidosis. J Am Coll Cardiol.

[14] Ruberg FL, Grogan M, Hanna M, Kelly JW, Maurer MS (2019). Transthyretin amyloid cardiomyopathy: JACC state-of-the-art review. J Am Coll Cardiol.

[15] Pagourelias ED, Mirea O, Duchenne J, Van Cleemput J, Delforge M, Bogaert J (2017). Echo parameters for differential diagnosis in cardiac amyloidosis: a head-to-head comparison of deformation and nondeformation parameters. Circ Cardiovasc Imaging.

[16] Remenyi B, Wilson N, Steer A, Ferreira B, Kado J, Kumar K (2012). World heart federation criteria for echocardiographic diagnosis of rheumatic heart disease--an evidence-based guideline. Nat Rev Cardiol.

[17] de Gregorio C, Dattilo G, Casale M, Terrizzi A, Donato R, Di Bella G (2016). Left atrial morphology, size and function in patients with transthyretin cardiac amyloidosis and primary hypertrophic cardiomyopathy- comparative strain imaging study. Circ J.

[18] Arvidsson S, Henein MY, Wikstrom G, Suhr OB, Lindqvist P (2018). Right ventricular involvement in transthyretin amyloidosis. Amyloid..

[19] Saito M, Wake D, Higaki R, Sakaue T, Morioka H, Sumimoto T, Inaba S (2019). Prognostic value of relative apical sparing pattern in patients with generalized left ventricular hypertrophy. JACC Cardiovasc Imaging.

[20] DeLong ER, DeLong DM, Clarke-Pearson DL (1988). Comparing the areas under two or more correlated receiver operating characteristic curves: a nonparametric approach. Biometrics..

[21] Dorbala S, Cuddy S, Falk RH (2020). How to image cardiac amyloidosis: a practical approach. JACC Cardiovasc Imaging.

[22] Maurer MS, Bokhari S, Damy T, Dorbala S, Drachman BM, Fontana M (2019). Expert consensus recommendations for the suspicion and diagnosis of transthyretin cardiac amyloidosis. Circ Heart Fail.

[23] Felker GM, Thompson RE, Hare JM, Hruban RH, Clemetson DE, Howard DL, Baughman KL, Kasper EK (2000). Underlying causes and long-term survival in patients with initially unexplained cardiomyopathy. N Engl J Med.

[24] Boldrini M, Cappelli F, Chacko L, Restrepo-Cordoba MA, Lopez-Sainz A, Giannoni A, Aimo A, Baggiano A, Martinez-Naharro A, Whelan C, Quarta C, Passino C, Castiglione V, Chubuchnyi V, Spini V, Taddei C, Vergaro G, Petrie A, Ruiz-Guerrero L, Moñivas V, Mingo-Santos S, Mirelis JG, Dominguez F, Gonzalez-Lopez E, Perlini S, Pontone G, Gillmore J, Hawkins PN, Garcia-Pavia P, Emdin M, Fontana M (2020). Multiparametric echocardiography scores for the diagnosis of cardiac amyloidosis. JACC Cardiovasc Imaging.

[25] Ternacle J, Bodez D, Guellich A, Audureau E, Rappeneau S, Lim P, Radu C, Guendouz S, Couetil JP, Benhaiem N, Hittinger L, Dubois-Randé JL, Plante-Bordeneuve V, Mohty D, Deux JF, Damy T (2016). Causes and consequences of longitudinal LV dysfunction assessed by 2D strain echocardiography in cardiac amyloidosis. JACC Cardiovasc Imaging.

[26] Aimo A, Chubuchny V, Vergaro G, Barison A, Nicol M, Cohen-Solal A (2021). A simple echocardiographic score to rule out cardiac amyloidosis. Eur J Clin Investig.

