# Supplementary material for: Cardiac amyloidosis screening using a relative apical sparing pattern in patients with left ventricular hypertrophy
Source: Cardiovasc Ultrasound. 2021 Aug 23;19:30. doi: 10.1186/s12947-021-00258-x (PMC8383373; doi:10.1186/s12947-021-00258-x)
Supplement: Supplementary file 1 — Additional file 1 Supplemental Method 1: Clinical data. Supplemental Method 2: Electrocardiogram (ECG). Supplemental Method 3: Standard transthoracic echocardiography. Supplemental Method 4: Strain imaging. Supplemental Table 1: Incremental benefits of continuous echocardiographic parameters over the base model. Supplemental Table 2: Baseline characteristics of patients with and without cardiac amyloidosis in the validation cohort. Supplemental Table 3: Reliability data. [file 12947_2021_258_MOESM1_ESM.docx]

**Supplementary Method 1: Clinical data**

Data (demographic data, comorbidities, medical history, laboratory data, imaging data, biopsy findings, and follow-up data) from the clinically stable condition at the closest time to echocardiography were collected by chart review.

**Supplementary Method 2: Electrocardiogram (ECG)**

A 12-lead ECG was used to assess conduction abnormalities, PQ, QRS, and heart-rate-corrected QT duration, low voltage (QRS amplitude <5 mm in frontal derivation and <10 mm in the precordial leads), pseudoinfarct pattern (QS wave pattern in two contiguous leads in the absence of previous myocardial infarction), and Sokolow-Lyon index (sum of the S wave in V_1_ lead and R wave in V_5_) (1,2).

**References**

1. Murtagh B, Hammill SC, Gertz MA, Kyle RA, Tajik AJ, Grogan M. Electrocardiographic findings in primary systemic amyloidosis and biopsy-proven cardiac involvement. Am J Cardiol 2005;95:535–7.

2. Sokolow M, Lyon TP. The ventricular complex in left ventricular hypertrophy as obtained by unipolar precordial and limb leads. Am Heart J 1949;37:161–86.

**Supplementary Method 3: Standard transthoracic echocardiography**

It was performed by experienced sonographers using a commercially available ultrasound system (Vivid 7 or Vivid E9 or Vivid E95; GE Vingmed, Horten, Norway.) The median duration between echocardiography and detailed work-up (biopsy, ^99m^Tc-PYP scintigraphy, or CMR) was 11 (IQR, 1–51) days. Standard two-dimensional greyscale still and moving images were recorded while three cardiac cycles. All echocardiographic data were transmitted on EchoPAC software (EchoPAC PC BT13: GE Healthcare) for subsequent offline analyses. Conventional echocardiographic measurements were analyzed at each participating hospital according to the recommendations of the American Society of Echocardiography (1–3). LV mass was calculated according to the American Society of Echocardiography formula and normalized to body surface area. LV volumes and LV ejection fraction were calculated with the biplane method of disks using two-dimensional images, and LV volumes were indexed to body surface area. Left atrial (LA) volume was calculated with the biplane-modified Simpson method using two-dimensional images and indexed to body surface area. The transmitral early diastolic velocity (E) and its deceleration time were obtained in the apical four-chamber view using a pulsed-wave Doppler at the level of the mitral valve tip during diastole. The early diastolic mitral annular tissue velocity (e’) was calculated as the septal mitral annular velocities, and E/e’ was calculated.

The following parameters, which were relatively specific to CA, were also assessed by a blinded sonographer at Kitaishikai Hospital. Granular sparkling—increased echogenicity of the thickened myocardium—was visually assessed (4). Presence of pericardial effusion was defined as >5 mm of circumferential pericardial separation during diastole (5). Anterior mitral valve leaflet thickness was measured at the thickest portion of the leaflet on a frame with maximal separation of chordae from the leaflet tissue after zooming during diastole (1,6). Maximum interatrial septal thickness was measured in the apical four-chamber view after zooming in the end-diastolic phase (7). Right ventricular wall thickness was measured using two-dimensional echocardiography from the left parasternal long-axis window after zooming at end-diastole (8).

**References**

1. Zoghbi WA, Enriquez-Sarano M, Foster E, et al. Recommendations for evaluation of the severity of native valvular regurgitation with two-dimensional and Doppler echocardiography. J Am Soc Echocardiogr 2003;16:777–802.

2. Lang RM, Badano LP, Mor-Avi V, et al. Recommendations for cardiac chamber quantification by echocardiography in adults: an update from the American Society of Echocardiography and the European Association of Cardiovascular Imaging. J Am Soc Echocardiogr 2015;28:1–39 e14.

3. Nagueh SF, Smiseth OA, Appleton CP, et al. Recommendations for the evaluation of left ventricular diastolic function by echocardiography: An update from the American Society of Echocardiography and the European Association of Cardiovascular Imaging. J Am Soc Echocardiogr 2016;29:277-314.

4. Siqueira-Filho AG, Cunha CL, Tajik AJ, Seward JB, Schattenberg TT, Giuliani ER. M-mode and two-dimensional echocardiographic features in cardiac amyloidosis. Circulation 1981;63:188–96.

5. Jung HO. Pericardial effusion and pericardiocentesis: role of echocardiography. Korean Circ J 2012;42:725–34.

6. Remenyi B, Wilson N, Steer A, et al. World Heart Federation criteria for echocardiographic diagnosis of rheumatic heart disease--an evidence-based guideline. Nat Rev Cardiol 2012;9:297–309.

7. Lim HE, Na JO, Im SI et al. Interatrial septal thickness as a marker of structural and functional remodeling of the left atrium in patients with atrial fibrillation. Korean J Intern Med

2015;30:808–20.

8. Rudski LG, Lai WW, Afilalo J, et al. Guidelines for the echocardiographic assessment of the right heart in adults: a report from the American Society of Echocardiography endorsed by the European Association of Echocardiography, a registered branch of the European Society of Cardiology, and the Canadian Society of Echocardiography. J Am Soc Echocardiogr 2010;23:685–713.

**Supplementary Method 4: Strain imaging**

Offline speckle-tracking analysis was performed on raw data with a dedicated software (EchoPAC PC BT13: GE Healthcare) by a blinded sonographer at Kitaishikai Hospital. Acquisition frame rates were 40–80 frames/s. The global longitudinal strain (GLS) was measured by averaging all segmental peak strain values in the apical four-chamber, two-chamber, and long-axis views (1). Strain values for the 6 basal, 6 mid, and 5 apical segments of LV were averaged to obtain regional longitudinal strain (LS) values (basal, mid, and apical, respectively). Additionally, the ejection fraction strain ratio was calculated as the LV ejection fraction divided by the absolute value of GLS. Left atrial (LA) strain was calculated as longitudinal strain obtained from a non-foreshortened apical four-chamber view. Tracing was manually adjusted to avoid interference of empty areas, including pulmonary vein ostia. According to the recent recommendation of atrial deformation imaging, ventricular end-diastole was set as the zero-baseline for the atrial strain curve (2). The LA reservoir and booster strains were measured as the positive peak strain during the reservoir and positive peak strains during contraction phase, respectively.

**References**

1. Mor-Avi V, Lang RM, Badano LP, et al. Current and evolving echocardiographic techniques for the quantitative evaluation of cardiac mechanics: ASE/EAE consensus statement on methodology and indications endorsed by the Japanese Society of Echocardiography. J Am Soc Echocardiogr 2011;24:277–313.

2. Badano LP, Kolias TJ, Muraru D, et al. Standardization of left atrial, right ventricular, and right atrial deformation imaging using two-dimensional speckle tracking echocardiography: a consensus document of the EACVI/ASE/Industry Task Force to standardize deformation imaging. Eur Heart J Cardiovasc Imaging 2018;19:591–600.

**Supplementary Table 1. Incremental benefits of continuous echocardiographic parameters over base model**

|  | **AUC (95%CI)** | **p value**  (base model vs base model plus each echo parameter) |
| --- | --- | --- |
| **Base model**: Age (≥65 [men], ≥70 [women])  + Low voltage+ PWT ≧14.0 mm | 0.82 (0.76–0.88) |  |
| + LV ejection fraction | 0.83 (0.76–0.90) | 0.62 |
| + E/e' | 0.86 (0.80–0.92) | **< 0.01** |
| + LA volume index | 0.83 (0.76–0.89) | 0.51 |
| + Interatrial septal wall thickness | 0.79 (0.71–0.87) | 0.06 |
| + Right ventricular wall thickness | 0.82 (0.75–0.89) | 0.95 |
| + Anterior mitral valve leaflet thickness | 0.82 (0.75–0.88) | 0.77 |
| + GLS | 0.83 (0.76–0.90) | 0.27 |
| + Ejection fraction strain ratio | 0.84 (0.77–0.90) | 0.12 |
| + LA reservoir strain | 0.89 (0.84–0.94) | **< 0.01** |
| + qRASP | 0.90 (0.85–0.95) | **< 0.01** |

AUC indicates area under the curve; CI, confidence interval; GLS, global longitudinal strain; LA, left atrial; LV, left ventricular; PWT, posterior wall thickness; qRASP, quantitatively assessed relative apical sparing pattern of longitudinal strain.

**Supplementary Table 2. Baseline patients’ characteristics in patients with and without cardiac amyloidosis in validation cohort**

| **Variables** | **Available data** | **Overall  (n=178)** | **CA**  **(n=56)** | **Non-CA*** **(n=122)** | **p**  **(CA vs Non-CA)** |
| --- | --- | --- | --- | --- | --- |
| Age (years) | 178 | 70 (58–80) | 82 (75–88) | 64 (55–73) | **< 0.01** |
| Male sex, n (%) | 178 | 122 (69) | 40 (71) | 82 (67) | 0.61 |
| Body weight (kg) | 178 | 59.8 (50.1–68.0) | 52.7 (47.2–59.3) | 63.7 (54.1–72.3) | **< 0.01** |
| Body mass index (kg/m^2^) | 178 | 23.5 (20.6–25.9) | 21.8 (20.1–23.7) | 24.6 (21.5–26.2) | **< 0.01** |
| Systolic blood pressure (mmHg) | 178 | 126 (113–142) | 117 (105–134) | 131 (120–143) | **< 0.01** |
| Diastolic blood pressure (mmHg) | 178 | 72 (64–84) | 70 (54–79) | 73 (66–84) | **< 0.01** |
| Heart rate (/min) | 178 | 69 (60–77) | 70 (62–78) | 67 (58–75) | 0.09 |
| NYHA functional class at discharge (I/II/III/IV), n (%) | 178 | 90/39/34/15 (51/22/19/8) | 9/16/21/10 (16/29/38/18) | 81/23/13/5 (66/19/11/4) | **< 0.01** |
| **Comorbidities** |  |  |  |  |  |
| Hypertension, n (%) | 178 | 88 (49) | 20 (36) | 68 (56) | **0.02** |
| Diabetes, n (%) | 178 | 41 (23) | 12 (21) | 29 (24) | 0.85 |
| Dyslipidemia, n (%) | 178 | 50 (28) | 8 (14) | 42 (34) | **< 0.01** |
| Atrial fibrillation, n (%) | 178 | 50 (28) | 12 (21) | 17 (16) | 0.28 |
| Device (N/PPM/ICD/CRT), n (%) | 178 | 149/8/12/9 (84/4/7/5) | 46/3/5/2 (82/5/9/4) | 103/5/7/7 (84/4/6/6) | 0.79 |
| **Serum markers** |  |  |  |  |  |
| B-type natriuretic peptide (pg/mL) | 164 | 191.4 (85.1–380.6) | 305.8 (198.7–649.5) | 131.7 (58.3–287.1) | **< 0.01** |
| Troponin positive†, n (%) | 111 | 73 (66) | 32 (91) | 41 (53) | **< 0.01** |
| Hemoglobin (g/L) | 171 | 13.5 (12.1–15.1) | 12.4 (11.4–14.1) | 14.0 (12.9–15.2) | **< 0.01** |
| eGFR (mL/min/1.73 m^2^) | 172 | 59.3 (46.6–74.3) | 51.4 (34.9–63.7) | 62.7 (50.7–76.7) | **< 0.01** |
| Sodium (mmol/L) | 171 | 140 (138–141) | 139 (136–141) | 140 (139–141) | 0.07 |
| Serum albumin (mg/L) | 157 | 4.0 (3.5–4.2) | 3.7 (3.5–4.1) | 4.1 (3.7–4.3) | **< 0.01** |
| **Electrocardiographic variables** |  |  |  |  |  |
| SV_1_+RV_5_ voltage (mm) | 178 | 2.7 (1.6–3.5) | 2.0 (1.1–2.9) | 3.0 (2.0–4.1) | **< 0.01** |
| PQ duration (ms) | 178 | 182 (162–205) | 188 (170–226) | 176 (156–200) | **0.02** |
| QRS duration (ms) | 178 | 104 (92–132) | 96 (88–134) | 106 (95–128) | **0.04** |
| Heart-rate-corrected QT (ms) | 178 | 442 (424–465) | 441 (427–458) | 442 (423–468) | 0.85 |
| Right bundle branch block, n (%) | 170 | 28 (16) | 10 (20) | 18 (15) | 0.50 |
| Left bundle branch block, n (%) | 166 | 10 (6) | 4 (8) | 6 (5) | 0.50 |
| Pseudo-infarct pattern, n (%) | 168 | 17 (10) | 10 (20) | 7 (6) | **0.01** |
| Low voltage, n (%) | 168 | 19 (11) | 13 (25) | 6 (5) | **< 0.01** |
| **Conventional echocardiographic variables** |  |  |  |  |  |
| Interventricular septal thickness (mm) | 176 | 12.1 (11.0–15.0) | 13.0 (11.8–15.2) | 12.0 (11.0–14.0) | **0.04** |
| LV posterior wall thickness (mm) | 176 | 11.0 (9.9–13.0) | 13.0 (10.7–14.4) | 10.5 (9.4–12.0) | **< 0.01** |
| LV mean wall thickness (mm) | 175 | 11.5 (10.5–14.0) | 13.2 (11.3–14.9) | 11.0 (10.4–13.0) | **< 0.01** |
| LV mass index (g/m^2^) | 175 | 133.7 (111.9–160.4) | 135.5 (111.8–172.0) | 131.7 (112.9–153.5) | 0.43 |
| LV end-diastolic diameter (mm) | 176 | 46.0 (42.6–52.0) | 43.0 (39.9–47.5) | 48.2 (44.0–54.0) | **< 0.01** |
| LV end-systolic diameter (mm) | 176 | 32.0 (26.7–37.9) | 32.1 (28.3–37.4) | 31.6 (26.0–38.0) | 0.61 |
| LV end-diastolic volume (mL) | 177 | 76.0 (61.0–107.2) | 71.4 (49.7–91.3) | 82.5 (62.7–125.3) | **< 0.01** |
| LV end-systolic volume (mL) | 177 | 30.0 (21.0–51.9) | 32.8 (22.9–46.1) | 30.0 (21.0–55.0) | 0.78 |
| LV ejection fraction (%) | 177 | 60.0 (50.0–66.1) | 54 (43–61) | 61 (52–67) | **< 0.01** |
| E velocity deceleration time (ms) | 178 | 190 (156–246) | 174 (145–202) | 207 (164–262) | **< 0.01** |
| e’-wave velocity (cm/s) | 175 | 4.3 (3.2–6.1) | 3.3 (2.7–4.2) | 5.1 (3.8–6.4) | **< 0.01** |
| Septal E/e’ | 175 | 16.1 (11.1–22.6) | 22.8 (19.2–29.7) | 13.8 (9.6–20.1) | **< 0.01** |
| LA volume index (mL/m^2^) | 174 | 46.9 (32.4–58.8) | 53.2 (39.9–61.2) | 40.6 (31.3–58.3) | **< 0.01** |
| Moderate to severe mitral regurgitation, n (%) | 178 | 17 (10) | 5 (9) | 12 (10) | 1.00 |
| Moderate to severe aortic regurgitation, n (%) | 178 | 19 (11) | 10 (18) | 9 (7) | 0.06 |
| Pericardial effusion, n (%) | 175 | 31 (18) | 14 (25) | 17 (14) | 0.09 |
| Granular sparkling, n (%) | 175 | 22 (13) | 13 (24) | 9 (8) | **< 0.01** |
| Anterior mitral valve leaflet thickness (mm) | 175 | 4.0 (3.3–4.6) | 2.8 (2.4–3.4) | 3.2 (2.7–3.7) | **< 0.01** |
| Interatrial septal wall thickness (mm) | 173 | 7.4 (6.1–8.6) | 7.5 (6.0–8.6) | 7.4 (6.2–8.6) | 0.94 |
| Right ventricular wall thickness (mm) | 175 | 4.0 (2.6–3.6) | 3.7 (3.3–4.6) | 4.0 (3.4–4.6) | 0.53 |
| **Strain imaging variables** |  |  |  |  |  |
| LV global longitudinal strain (%) | 174 | -10.8 (-14.4–-8.2) | -9.9 (-12.4–-8.2) | -11.3 (-15.0–-8.2) | 0.06 |
| Ejection fraction strain ratio | 174 | 5.1 (4.0–6.5) | 5.3 (4.3–6.7) | 5.0 (3.8–6.3) | 0.35 |
| LA longitudinal strain (reservoir phase) (%) | 159 | 13.1 (6.9–19.9) | 7.5 (5.2–11.1) | 16.0 (10.4–23.2) | **< 0.01** |
| LA longitudinal strain (booster phase) (%) | 129 | 6.9 (3.6–11.0) | 3.9 (1.9–5.5) | 9.4 (4.9–12.0) | **< 0.01** |
| qRASP | 174 | 0.73 (0.55–0.96) | 1.03 (0.78–1.23) | 0.65 (0.50–0.78) | **< 0.01** |
| qRASP >1.00, n (%) | 174 | 38 (21) | 32 (57) | 6 (5) | **< 0.01** |
| qRASP >0.90, n (%) | 174 | 49 (28) | 35 (63) | 14 (12) | **< 0.01** |
| qRASP >0.87, n (%) | 174 | 53 (30) | 36 (64) | 17 (14) | **< 0.01** |
| sRASP, n (%) | 174 | 46 (26) | 32 (57) | 14 (12) | **< 0.01** |

Data are expressed as the median (interquartile range) or number (percentage).

* The etiology of left ventricular hypertrophy in the remaining 122 patients without CA was hypertrophic cardiomyopathy (n=56), hypertensive heart disease (n=40), dilated cardiomyopathy (n=13), cardiac sarcoidosis (n=7), valvular heart disease (n=4), and Fabry disease (n=2).

†Troponin I (n = 111) ≥ 26.2 pg/mL. Troponin T was not available.

ACEi indicates angiotensin-converting-enzyme inhibitor; AF, atrial fibrillation; ARB, angiotensin receptor blocker; CA, cardiac amyloidosis; CRT, cardiac resynchronization therapy; ICD, implantable cardioverter-defibrillator; LA, left atrial; LV, left ventricular; NYHA, New York Heart Association; PPM, permanent pacemaker; qRASP, quantitatively assessed relative apical sparing pattern of longitudinal strain; sRASP, semi-quantitatively assessed relative apical sparing pattern of longitudinal strain.

**Supplementary Table 3. Reliability data**

| **Variables** | **Interobserver variability** | | **Intraobserver variability** | |
| --- | --- | --- | --- | --- |
|  | **ICC (95% CI)** | **κ** | **ICC (95% CI)** | **κ** |
| Anterior mitral valve leaflet thickness | 0.31 (-0.05–0.61) | - | 0.27 (-0.07–0.55) | - |
| Interatrial septal wall thickness | 0.50 (0.20–0.71) | - | 0.64 (0.39–0.79) | - |
| Right ventricular wall thickness | 0.26 (-0.18–0.61) | - | 0.28 (-0.06–0.57) | - |
| LV global longitudinal strain | 0.94 (0.89–0.97) | - | 0.95 (0.91–0.98) | - |
| Ejection fraction strain ratio | 0.85 (0.70–0.92) | - | 0.88 (0.77–0.94) | - |
| LA longitudinal strain (reservoir phase) | 0.75 (0.57–0.87) | - | 0.84 (0.71–0.91) | - |
| LA longitudinal strain (booster phase) | 0.78 (0.61–0.88) | - | 0.82 (0.68–0.91) | - |
| qRASP | 0.92 (0.84–0.96) | - | 0.91 (0.84–0.95) | - |
| Pericardial effusion (>5mm) | - | 0.84 | - | 0.85 |
| Granular sparkling | - | 0.47 | - | 0.55 |
| sRASP | - | 0.92 | - | 0.93 |

CI indicates confidence interval; ICC, intraclass correlation coefficients; κ, the kappa statistic; LA, left atrial; LV, left ventricular; qRASP, quantitatively assessed relative apical sparing pattern of longitudinal strain; sRASP, semi-quantitatively assessed relative apical sparing pattern of longitudinal strain.
