# Supplementary figures and images for: Cardiac amyloidosis screening using a relative apical sparing pattern in patients with left ventricular hypertrophy
Source: Cardiovasc Ultrasound. 2021 Aug 23;19:30. doi: 10.1186/s12947-021-00258-x (PMC8383373; doi:10.1186/s12947-021-00258-x)

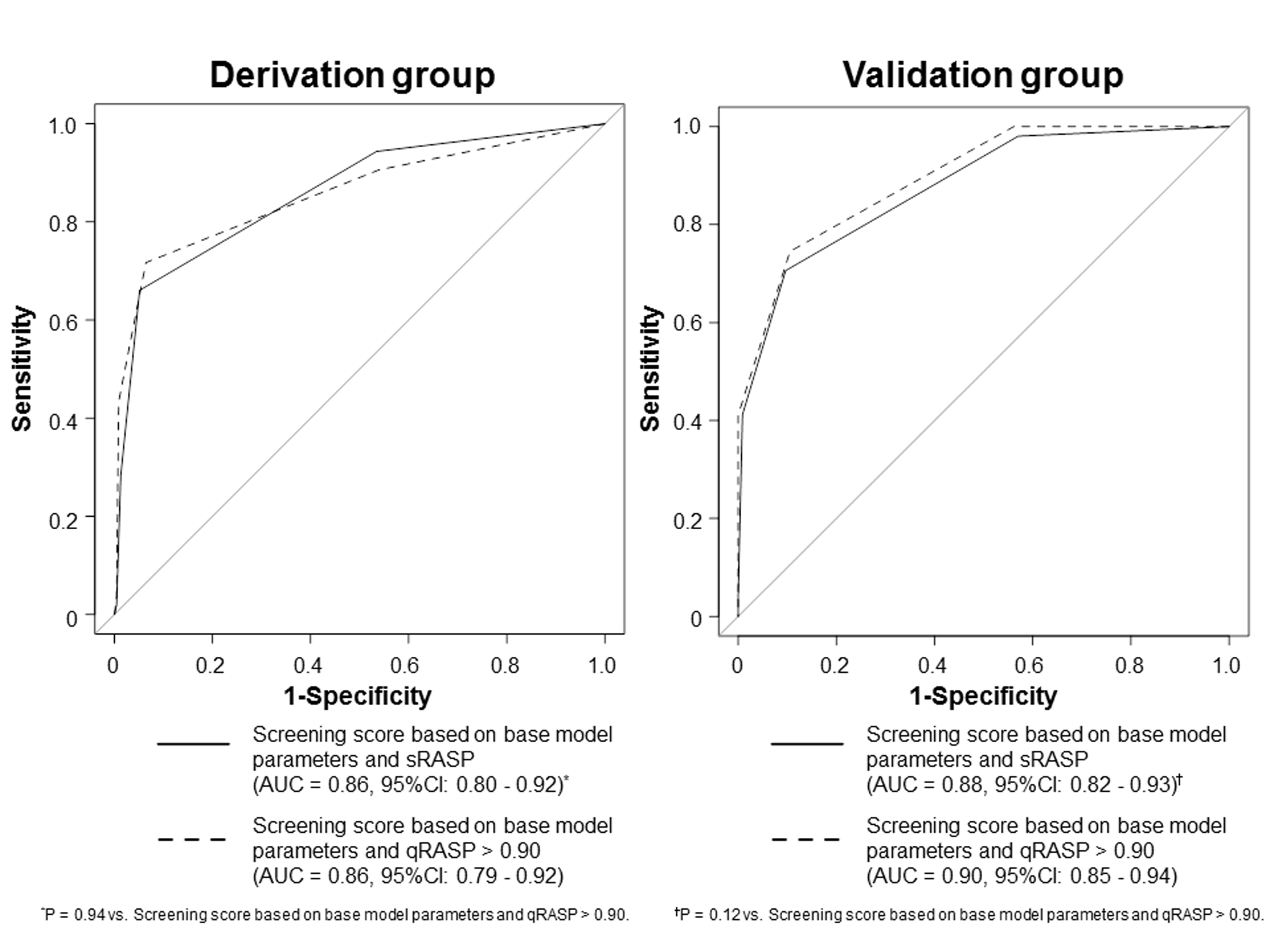

Supplement: Supplementary file 2 — Additional file 2 Supplemental Fig. 1: Comparison between the screening score based on base model parameters and semi-quantitatively assessed RASP and based on base model parameters and quantitatively assessed RASP > 0.90 in the derivation (left) and validation groups (right). AUC; area under the curve; CI, confidence interval; qRASP, quantitative relative apical sparing pattern; sRASP, semi-quantitative relative apical sparing pattern. [file 12947_2021_258_MOESM2_ESM.tif]

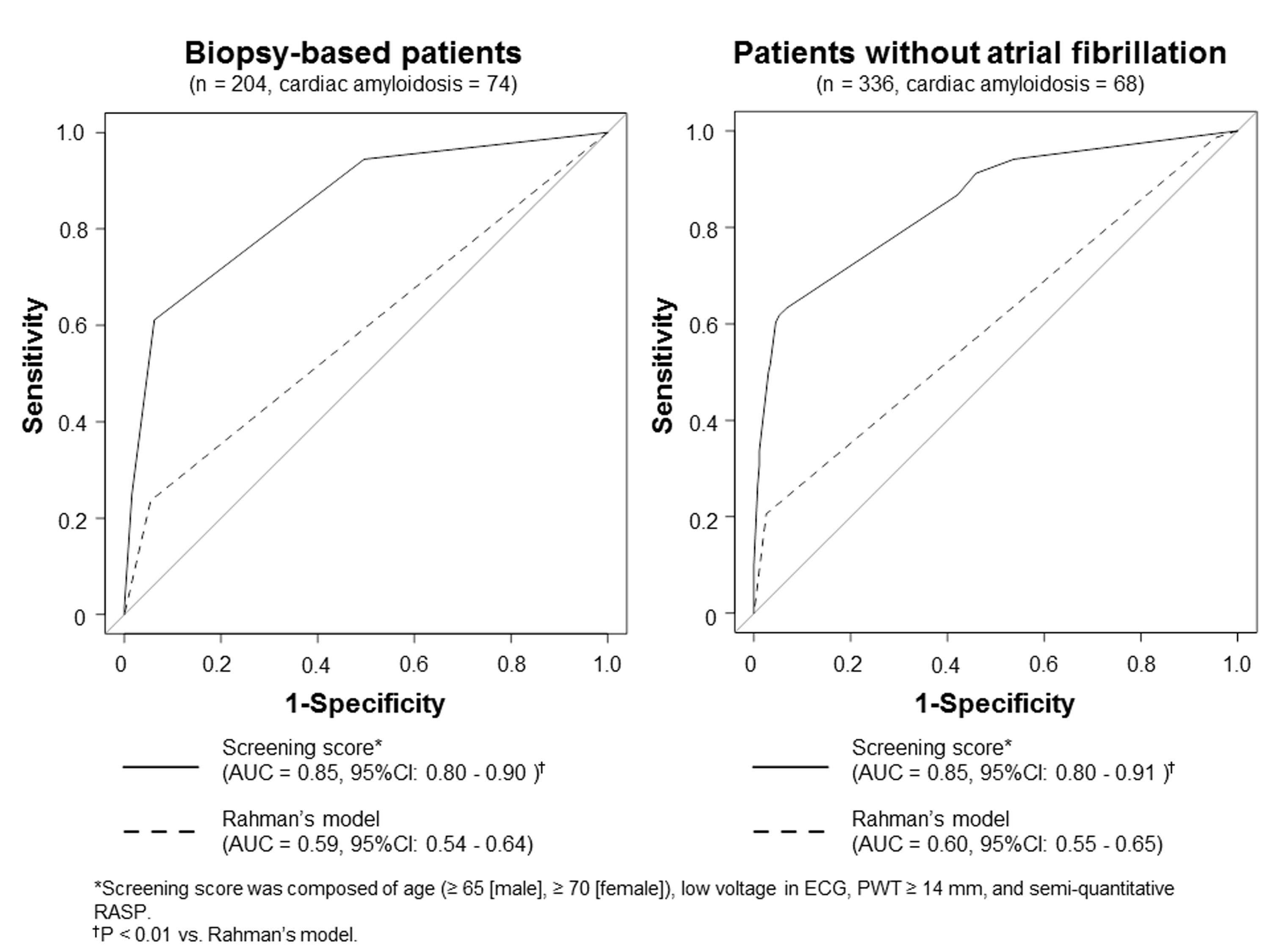

Supplement: Supplementary file 3 — Additional file 3 Supplemental Fig. 2: Comparison of the new screening score with Rahman’s model in the selected patients. AUC; area under the curve; CA, cardiac amyloidosis; CI, confidence interval; PWT, posterior wall thickness; RASP, relative apical sparing pattern. [file 12947_2021_258_MOESM3_ESM.tif]
